# Korean Red Ginseng Attenuates Particulate Matter-Induced Senescence of Skin Keratinocytes

**DOI:** 10.3390/antiox12081516

**Published:** 2023-07-28

**Authors:** Kyoung Ah Kang, Mei Jing Piao, Pincha Devage Sameera Madushan Fernando, Herath Mudiyanselage Udari Lakmini Herath, Joo Mi Yi, Jin Won Hyun

**Affiliations:** 1 Department of Biochemistry, College of Medicine, Jeju National University, Jeju 63243, Republic of Korea; legna07@jejunu.ac.kr (K.A.K.); mjpiao@jejunu.ac.kr (M.J.P.); sameera@stu.jejunu.ac.kr (P.D.S.M.F.); lakmini@stu.jejunu.ac.kr (H.M.U.L.H.); 2 Jeju Research Center for Natural Medicine, Jeju National University, Jeju 63243, Republic of Korea; 3 Department of Microbiology and Immunology, College of Medicine, Inje University, Busan 47392, Republic of Korea; jmyi76@inje.ac.kr

**Keywords:** fine particulate matter, skin cellular senescence, Korean red ginseng, epigenetic alteration

## Abstract

Skin is a direct target of fine particulate matter (PM_2.5_), as it is constantly exposed. Herein, we investigate whether Korean red ginseng (KRG) can inhibit PM_2.5_-induced senescence in skin keratinocytes. PM_2.5_-treated human keratinocyte cell lines and normal human epidermal keratinocytes showed characteristics of cellular senescence, including flat and enlarged forms; however, KRG suppressed them in both cell types. Moreover, while cells exposed to PM_2.5_ showed a higher level of p16^INK4A^ expression (a senescence inducer), KRG inhibited its expression. Epigenetically, KRG decreased the expression of the ten-eleven translocation (TET) enzyme, a DNA demethylase induced by PM_2.5_, and increased the expression of DNA methyltransferases suppressed by PM_2.5_, resulting in the decreased methylation of the *p16^INK4A^* promoter region. Additionally, KRG decreased the expression of mixed-lineage leukemia 1 (MLL1), a histone methyltransferase, and histone acetyltransferase 1 (HAT1) induced by PM_2.5_. Contrastingly, KRG increased the expression of the enhancer of zeste homolog 2, a histone methyltransferase, and histone deacetyltransferase 1 reduced by PM_2.5_. Furthermore, KRG decreased TET1, MLL1, and HAT1 binding to the *p16^INK4A^* promoter, corresponding with the decreased mRNA expression of p16^INK4A^. These results suggest that KRG exerts protection against the PM_2.5_-induced senescence of skin keratinocytes via the epigenetic regulation of *p16^INK4A^*.

## 1. Introduction

Air pollutants in cities represent a serious health problem, with fine particulate matter (PM_2.5_) accounting for a large portion, owing to coal combustion and diesel exhaust fumes [1]. Both indoors and outdoors, PM_2.5_ damages several human systems, including the cardiovascular, central nervous, and pulmonary immune systems [2,3,4,5]. PM_2.5_ mainly penetrates the skin barrier via the appendix pathway and stratum corneum, which can interfere with skin protection activities, resulting in wrinkles and thickening [6,7]. Furthermore, PM_2.5_ may induce both oxidative stress and inflammation, leading to skin aging [8]. Previously, we have reported that PM_2.5_ contributes to senescence in human keratinocytes through oxidative-stress-dependent epigenetic regulation [9].

Changes in the external environment, such as exposure to PM_2.5_, have been reported to affect gene expression through epigenetic regulation [10,11]; wherein, PM exposure leads to hypo-methylation in the promoters of genes involved in oxidative stress, inflammation, DNA repair, and cell cycle regulation.

In mammals, gene expression is strictly controlled by epigenetic modifications, such as DNA methylation and histone modifications. In general, the methylation of DNA via DNA methyltransferases (DNMT1, DNMT3A, and DNMT3B) induces transcriptional silence. However, three DNA demethylases (ten-eleven translocations, TETs: TET1, TET2, and TET3) can reverse this methylation process, leading to transcriptional activation [12,13]. In addition to DNA methylation, many post-translation variations in histones play an essential role in changing chromatin structures and gene expression. The methylation of histone lysine and arginine residues is a marker of gene regulation. Histone methylation is complex, because residues can be mono-, di-, or trimethylated, and these indications can activate or inhibit gene transcription [14]. Di- and trimethylation of H3K4, H3K36, and H3K79 by a histone methyltransferase (HMT) are strongly correlated with the active transcription of genes, while the methylation of H3K9 and H3K27 by other HMTs inhibits transcription [15]. Furthermore, the acetylation of histone-tailed lysine residue is another marker of gene regulation. Histone acetyltransferases (HATs) add negatively charged acetyl groups to histones. This destabilizes the electrostatic interactions between the DNA surrounding the histone core, leading to transcription activation. On the contrary, acetyl group removal of histone deacetyltransferase (HDAC) regenerates stabilized electrostatic interactions, thereby inhibiting transcription induction.

Cellular senescence is typically characterized by a large, flat cell morphology, senescence-associated heterochromatin foci (SAHF), and β-galactosidase activity [16]. The tumor repressor p16^INK4A^ is a major controller of cellular senescence and inhibits cell cycle progression through its action as a specific cyclin-dependent kinase (CDK) 4 and 6 inhibitor [17]. Recently, it has been suggested that widespread epigenetic changes in senescent cells are crucial for the induction, progression, and maintenance of senescence [18]. The expression of p16^INK4A^ mRNA is reduced via the methylation of DNA at its CpG promoter site [19,20,21]. Moreover, in nucleosomal histones, p16^INK4A^ expression is silenced by H3K27Me3 via the enhancer of zeste homolog 2 (EZH2), an HMT [22]. In addition, p16^INK4A^ expression can be suppressed by HDAC, which binds to the *p16^INK4A^* promoter and represses *p16^INK4A^* transcription [23]. In contrast, mixed-lineage leukemia 1 (MLL1), an HMT, binds to the *p16^INK4A^* promoter, promoting H3K4Me3 and activating p16^INK4A^ transcription during premature senescence [24]. Furthermore, HAT binds to the *p16^INK4A^* promoter and induces the transcription of p16^INK4A^ [25].

Ginseng (*Panax ginseng* C. A. Meyer) is regarded as a traditional medicine and is used as a nutritional supplement and for the treatment of various diseases in Asia, such as cancers, immune disorders, and liver diseases. Recent clinical studies have shown that Korean red ginseng (KRG) normalizes various antioxidant markers to suppress intracellular oxidative stress [26]. KRG extract has also been shown to regulate nuclear factor kappa B (NF-κB) activation, microRNA biogenesis, and the endothelial nitric oxide synthase/nitric oxide pathway, likely by stimulating the heme oxygenase/carbon monoxide pathway [27,28,29,30]. However, the epigenetic molecular mechanisms and effects of KRG on senescence in human keratinocytes remain uncertain. Therefore, herein, we have examined whether KRG could reduce the PM_2.5_-induced senescence of human keratinocytes via epigenetic regulation.

## 2. Materials and Methods

### 2.1. Reagents

The 3-(4,5-Dimethylthiazol-2-yl)-2,5-diphenyltetrazolium bromide (MTT; cat. M5655) was obtained from Sigma-Aldrich Co., Ltd. (St. Louis, MO, USA). The primary antibodies against p16^INK4A^ (cat. #80772), DNMT3A (cat. #2160) and EZH2 (cat. #5246), were obtained from Cell Signaling Technology (Danvers, MA, USA). The TET1 (cat. MA5-16312), MLL1 (cat. A300-374A), and TATA-binding protein (TBP; cat. MA1-189) antibodies were obtained from Thermo Fisher Scientific Inc. (Waltham, MA, USA). The DNMT1 (cat. ab87654), H3K27Me3 (cat. ab195477), and H3K4Me3 (cat. ab8580) antibodies were obtained from Abcam (Cambridge, UK). The TET2 (cat. sc-398535), TET3 (cat. sc-518126), DNMT3B (cat. sc-376043), and actin (cat. sc-47778) antibodies were obtained from Santa Cruz Biotechnology (Santa Cruz, CA, USA). The SPiDER-βGal kit was obtained from Dojindo Molecular Technologies (Rockville, MD, USA).

### 2.2. KRG Preparation

The KRG was prepared by the Korea Ginseng Corporation (Seoul, Republic of Korea) from the roots of 6-year-old P. ginseng plants, as described previously [31]. Subsequently, P. ginseng was steamed for 3 h at 90–100 °C, after which it was dried at 50–80 °C. The KRG was then extracted through water circulation for 8 h at 85–90 °C. The ginsenosides of KRG were compared to the ginsenoside standards using ultra-performance liquid chromatography. The prepared KRG mainly contained the following ginsenosides: Rb1 (4.62 mg/g), Rg2 (3.21 mg/g), Rg3 (3.05 mg/g), Rc (2.41 mg/g), Rb2 (1.83 mg/g), Rf (1.21 mg/g), Rd (0.89 mg/g), Re (0.93 mg/g), and Rg1 (0.71 mg/g). The KRG was dissolved in dimethyl sulfoxide (DMSO) with final DMSO concentrations of <0.1% treated into the cell medium.

### 2.3. PM_2.5_ Preparation

Standard diesel PM (SRM 1650b, Sigma-Aldrich Co., Ltd.), with an average diameter of 0.18 µm, was used, mostly comprising polycyclic aromatic hydrocarbons (PAHs) and nitro-PAHs. Information on nitro-PAHs and PAHs in SRM 1650b, such as the certified mass fraction values, can be found in previously published papers [32,33]. PM_2.5_ in DMSO was prepared for stock, with DMSO as the control and with concentrations not exceeding 0.01%.

### 2.4. Keratinocyte Culture

The human keratinocytes cell line HaCaT was obtained from the CLS Cell Lines Service (Eppelheim, Germany). Normal human epidermal keratinocytes (NHEK) were obtained from Thermo Fisher Scientific. The HaCaT cells were seeded using a complete DMEM medium with 10% fetal bovine serum and 1% antibiotics. NHEK cells were cultured using EpiLife serum-free medium with added EpiLife undefined growth supplements (Thermo Fisher Scientific). All cells were incubated at 37 °C in a 5% CO_2_ incubator.

### 2.5. Cell Viability

To detect the cytotoxicity effect of KRG, cells were seeded (1 × 10^5^ cells/mL) and treated with 2, 4, 6, 8, 10, 20, 40, 80, or 100 µg/mL of KRG. After 48 h, the cells were cocultured with MTT solution. After a four-hour-long incubation period, the transformed formazan crystals were dissolved in DMSO. The formazan product was measured at an absorbance of 540 nm under a scanning multi-well spectrophotometer (Thermo Fisher Scientific).

### 2.6. Detection of Intracellular Reactive Oxygen Species (ROS)

To measure the ROS scavenging effect of KRG, we proceeded with 2′,7′-dichlorofluorescein diacetate (DCF-DA; Sigma-Aldrich Co., Ltd.) staining. The cells were pre-treated with 2, 4, 6, 8, 10, or 20 µg/mL of KRG and then treated with PM_2.5_ (50 μg/mL) for 24 h, which generated ROS, followed by the addition of 25 µM of DCF-DA. The ROS levels were detected using flow cytometry (Becton Dickinson, Mountain View, CA, USA) [34].

### 2.7. Colony Formation Detection

To detect the colony-forming ability (CFA), cells were seeded to 5 × 10^2^ cells/mL in a 60-mm dish. The colony expansion of single cells was allowed to progress for 3 days, after which they were pre-treated with 20 μg/mL of KRG, and then 50 μg/mL of PM_2.5_ was added for 7 days. The colony formation detection was performed using a Diff-Quik kit (Sysmex Corporation, Kobe, Japan). The Diff-Quik staining solution was used to stain the resulting colonies after fixing with Diff-Quik fixation solution.

### 2.8. SAHF Detection

To measure SAHF, an indicator of cellular senescence, cells were seeded to 1.0 × 10^5^ cells/mL on microscope slides, pre-treated with 20 µg/mL of KRG, and then treated with PM_2.5_ (50 μg/mL) for 24 h. Subsequently, the cells were labeled on the nucleus by 4′,6-diamidino-2-phenylindole (DAPI) staining. Cells were imaged using a Zeiss confocal microscope and LSM 510 software version 4.2 (Carl Zeiss, Oberkochen, Germany).

### 2.9. Immunofluorescence

To measure the expression of epigenetic-related proteins, the cells were seeded to 1.0 × 10^5^ cells/mL on microscope slides, were pre-treated with 20 µg/mL of KRG, and then treated with PM_2.5_ (50 μg/mL) for 24 h. Subsequently, the cells were fixed with 4% paraformaldehyde for 15 min and permeabilized with a permeable reagent (phosphate-buffered saline, PBS, containing 0.1% Triton X-100) for 15 min. The cells were incubated with a blocking solution (PBS containing 3% BSA) for 1 h and then incubated with the primary antibody diluted in the blocking solution for 2 h. The secondary antibody was incubated for 1 h using Alexa488-binding secondary antibody (Santa Cruz Biotechnology). The cells were observed under a confocal microscope (Zeiss, LSM 510 software) after mounting with a DAPI-containing mounting medium.

### 2.10. β-Galactosidase Activity Detection

To measure β-galactosidase activity, an indicator of cellular senescence, the cells were seeded on microscope slides, pre-treated with 20 µg/mL of KRG, and then treated with PM_2.5_ (50 μg/mL) for 24 h. Subsequently, the cells were stained with SPiDER-βGal staining and then incubated at 37 °C for 15 min. After that, the cells were mounted using DAPI-containing mounting medium and images were captured using a confocal microscope.

### 2.11. Western Blot Analysis

The cells were seeded to 1.0 × 10^5^ cells/mL on a 60-mm culture dish. They were pre-treated with 20 µg/mL of KRG and then treated with PM_2.5_ (50 μg/mL) for 24 h. The cells were collected, and the proteins were extracted by lysis. Subsequently, the lysates of cells (60 μg of protein) were separated via 6, 10, or 12% SDS-polyacrylamide gel electrophoresis and then transferred into the membrane, which was immunoblotted with specific primary antibodies. The following primary antibodies were used; p16^INK4A^, DNMT1, DNMT3A, DNMT3B, TET1, TET2, TET3, EZH2, H3K27Me3, MLL1, H3K4Me3, HDAC1, and HAT1. In addition, primary antibodies against actin and TBP were used as a loading control. Membranes bound with primary antibodies were contacted with secondary antibodies (Pierce, Rockland, IL, USA), and bands of protein were assessed using a western blotting detection kit (Amersham, Little Chalfont, Buckinghamshire, UK).

### 2.12. Quantitative Reverse Transcription Polymerase Chain Reaction (qRT-PCR)

The cells were seeded to 1.0 × 10^5^ cells/mL on a 60-mm culture dish. They were pre-treated with 20 µg/mL of KRG and then treated with PM_2.5_ (50 μg/mL) for 24 h. For the real-time qRT-PCT, the qRT-PCR reaction system contained 5.0 μL of 2× SYBR Green Mixture, forward primer (5 μM), and reverse primer (5 μM) each, 1.0 μL of cDNA, and double-distilled water. The qRT-PCR conditions were as follows: pre-denatured for 10 min at 95 °C; 40 cycles at 95 °C for 15 s; and at 60 °C for 1 min on a Bio-Rad iQ5 Real-Time PCR Detection System (Bio-Rad Laboratories, Hercules, CA, USA). The qRT-PCR primers used were as follows: forward primer 5′-CTCGTGCTGATGCTACTGAGGA-3′ and reverse primer 5′-GGTCGGCGCAGTTGGGCTCC-3′ for the amplification of p16^INK4A^ and forward primer 5′-CACCTTCTACAATGAGCTGCGTGT-3′ and reverse primer 5′-CACAGCCTGGATAGCAACGTACA-3′ for actin. A prepared 1% agarose gel with ethidium bromide was used to resolve the amplification, and it was photographed under ultraviolet light using Image Quant™ TL analysis software version 10.1 (Amersham Bioscience, Uppsala, Sweden). 

### 2.13. Quantitative Methylation-Specific PCR (qMSP) and Bisulfite Sequencing

To measure the methylation of the p16^INK4A^ promoter, a specific methylation kit (Zymo Research, Tustin, CA, USA) was used for DNA (1 μg) conversion, which involves the chemical bisulfite conversion of unmethylated cytosine to uracil and the protection of methylated cytosine. For qMSP detection, qMSP primer pairs were used to assess gene promoter methylation sites, which are located close to the putative transcription start site of the 5′ CpG islands. The qMSP was carried out using bisulfite-treated samples, was normalized based on Alu element amplification, and was assessed using the CFX96^TM^ real-time system (Bio-Rad Laboratories). In addition, for bisulfite sequencing, bisulfite-treated DNA and JumpStart REDTaq DNA polymerase (Sigma-Aldrich Co., Ltd.) were used for the template and amplification, respectively. A gel extraction kit (Qiagen GmbH, Hilden, Germany) and clones using the TOPO TA vector system (Invitrogen, Carlsbad, CA, USA) were used to purify the PCR products in bisulfite sequencing. Then, a NucleoSpin plasmid isolation kit (Macherey-Nagel, Düren, Germany) was used for the isolation and purification of each clone. An M13F primer was used to sequence the randomly selected positive clones. After that, the methylation status of each CpG dinucleotide was examined. qMSP and bisulfite sequencing primers were obtained based on previous reports [9,35,36].

### 2.14. Chromatin Immunoprecipitation (ChIP) Assay

To measure the binding of epigenetic-related proteins to the p16^INK4A^ locus, the cells were pre-treated with 20 µg/mL of KRG and then treated with PM_2.5_ (50 μg/mL) for 24 h. Antibodies against TET1, DNMT1, EZH2, MLL1, HDAC1, HAT1, and normal rabbit immunoglobulin G (IgG) were used for ChIP assays, which were assessed using a SimpleChIP ^®^ enzymatic ChIP kit (Cell Signaling Technology). DNA (200 ng) recovered from the immunoprecipitated complexes was subjected to qPCR. Primers for the p16^INK4A^ locus have been described previously [9]. The forward primer used was 5′-CCCCTTGCCTGGAAAGATAC-3′ and the reverse primer was 5′-AGCCCCTCCTCTTTCTTCCT-3′.

### 2.15. Transfection of Small Interfering RNA (siRNA)

Control siRNA (SS-1001), siRNA, TET1#1 sense (5′-CAGUGUAACCAGCACAGUU-3′) and antisense (5′-AACUGUGCUGGUUACACUG-3′) siRNAs, MLL1#1 sense (5′-GUCACAGUAGGUGAUCCUU-3′) and antisense (5′-AAGGAUCACCUACUGUGAC-3′) siRNAs, MLL1#2 sense (5′-CUAUUCUCGGGUCAUCAAU-3′) and antisense (5′-AUUGAUGACCCGAGAAUAG-3′) siRNAs, and HAT1#1 sense (5′-CUAUUCUCGGGUCAUCAAU-3′) and antisense (5′-AUUGAUGACCCGAGAAUAG-3′) siRNAs were obtained from Bioneer Corporation (Daejeon, Republic of Korea). TET1#2 (AM16708-ID:147892) and HAT1#2 (AM16708-ID:13322) siRNAs were obtained from Invitrogen. The cells were transfected using serum-free Opti-MEM containing Lipofectamine RNAiMax reagent (Invitrogen). Subsequently, we added 4 μL of Lipofectamine to 2 mL of Opti-MEM and let the mixture settle at 18–20 °C for 5 min. The siRNA was then added to the Opti-MEM-lipofectamine solution at a final concentration of 20 nM, after which the mixture was kept at 18–20 °C for 5 min. The transfection incubation was conducted for 24 h.

### 2.16. Statistical Analysis

Each experiment was repeated three times, and the results are indicated as the mean ± standard error of the mean (SEM). For the statistical analysis, SigmaStat software v12 (SPSS, Chicago, IL, USA) was used. One-way analysis of variance (ANOVA) and Tukey’s post hoc test were used to assess the results. At *p* < 0.05, differences were considered statistically significant.

## 3. Results

### 3.1. KRG Decreased ROS Generation Induced by PM_2.5_

**Figure 1:**
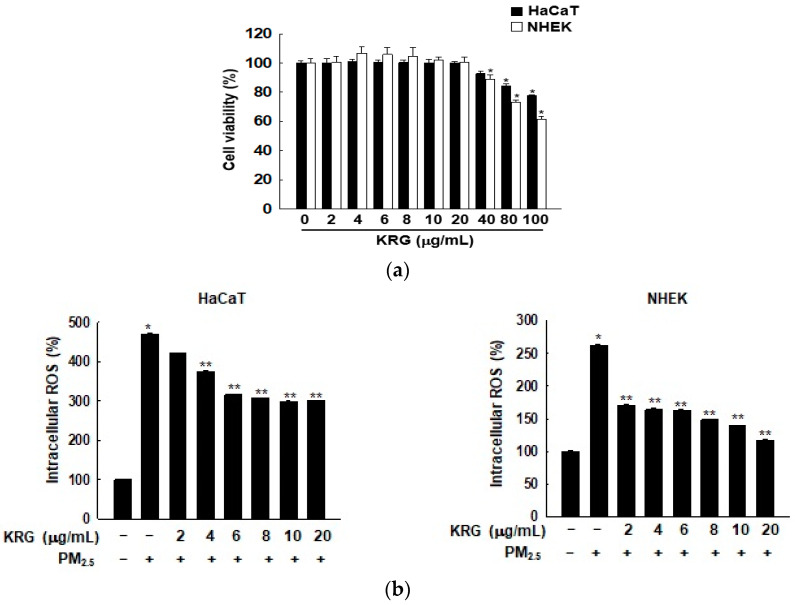
Scavenging effect of Korean red ginseng (KRG) against reactive oxygen species (ROS) generated by PM_2.5_. (**a**) Cell viability after treatment with indicated concentrations of KRG in HaCaT and normal human epidermal keratinocyte (NHEK) cells was assessed. (**b**) Intracellular ROS were measured by flow cytometry. * *p* < 0.05 indicates significant differences with untreated cells and ** *p* < 0.05 indicates significant differences with PM_2.5_-treated cells.

Previously, we have demonstrated that 50 μg/mL of PM_2.5_ exhibited ROS-induced senescence [9]; therefore, the optimal concentration of PM_2.5_ to include cellular senescence was set as 50 μg/mL. We first estimated the cell viability at various concentrations of KRG by using HaCaT and NHEK cells, finding that KRG showed no cytotoxicity up to doses of 20 μg/mL (Figure 1a). The ROS scavenging effect of KRG on ROS generation induced by PM_2.5_ in HaCaT and NHEK cells was highest at 20 μg/mL of KRG (Figure 1b). Therefore, 20 μg/mL of KRG was used as the optimal concentration for the subsequent analyses.

### 3.2. KRG Attenuated Cellular Senescence Induced by PM_2.5_

**Figure 2:**
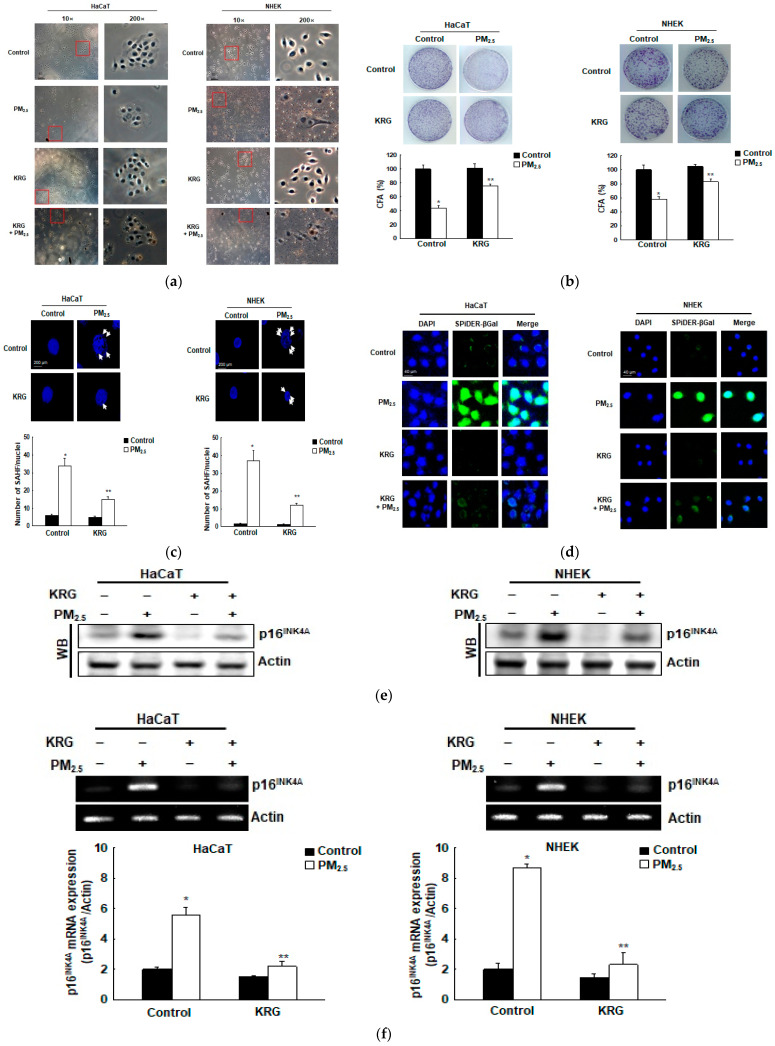
Inhibitory effect of KRG against cellular senescence induced by PM_2.5_. (**a**) Microscopic assessment reveals that PM_2.5_ induces morphological alterations typical of cellular senescence. Red square area means enlarged cell morphology. (**b**) Colony-forming ability (CFA) was enumerated after Diff-Quik staining. (**c**) Senescence-associated heterochromatin foci (SAHF) structure after cell staining with 4′,6-diamidino-2-phenylindole (DAPI) fluorescent dye was assessed. The arrows indicate SAHF in senescent cells induced by PM_2.5_. * *p* < 0.05 and ** *p* < 0.05 indicate significant differences with control cells and PM_2.5_-exposed cells, respectively. (**d**) β-galactosidase activity was revealed by staining with DAPI (blue) and the SPiDER-βGal (green) working solution. (**e**) Senescence marker p16^INK4A^ was detected by western blotting using the corresponding antibodies after cell lysates were electrophoresed. Actin represents a loading control. (**f**) Expression of p16^INK4A^ mRNA was assessed by qRT-PCR. * *p* < 0.05 and ** *p* < 0.05 indicate significant differences with control cells and PM_2.5_-exposed cells, respectively.

Oxidative stress induced by PM_2.5_ significantly showed senescence phenotypes in cells, such as an irregular size, a flattened and enlarged cell shape (Figure 2a), reduced CFA (Figure 2b), increased SAHF in the nucleus (Figure 2c), and higher cytoplasmic β-galactosidase activity (Figure 2d). However, the KRG treatment nearly restored the normal cell shape (Figure 2a), increased the CFA (Figure 2b), decreased the SAHF (Figure 2c), and reduced the β-galactosidase activity (Figure 2d). The expression of the p16^INK4A^ protein, a well-known CDK inhibitor and senescence inducer, was strongly induced in the PM_2.5_-treated cells, compared with that in the control cells; however, KRG treatment reduced its expression in both of the cell types (Figure 2e). In agreement with the western blotting results, p16^INK4A^ mRNA in the PM_2.5_-treated cells was also induced, compared with that in the control cells; however, KRG application reduced p16^INK4A^ mRNA (Figure 2f).

### 3.3. p16^INK4A^ Expression in KRG-Treated Cells Was Attenuated through Decreased DNA Demethylation in Cellular Senescence Induced by PM_2.5_

**Figure 3:**
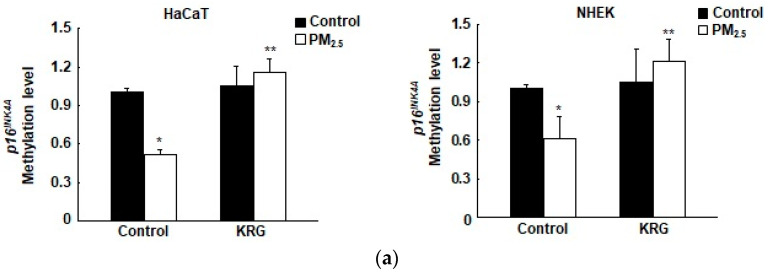

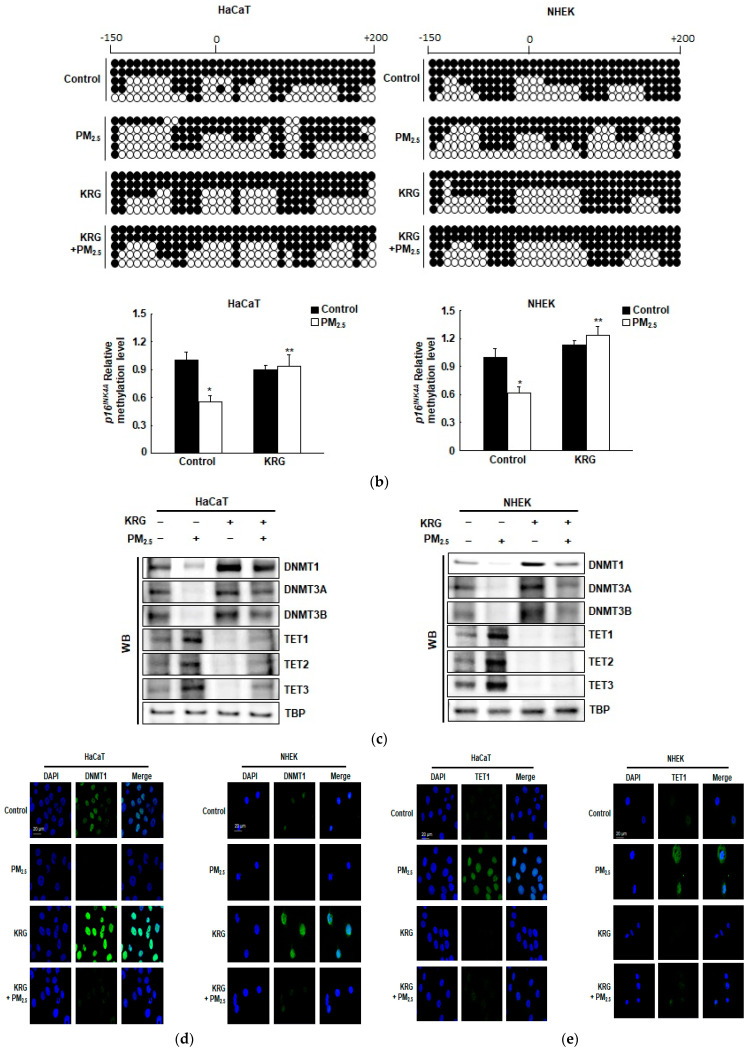

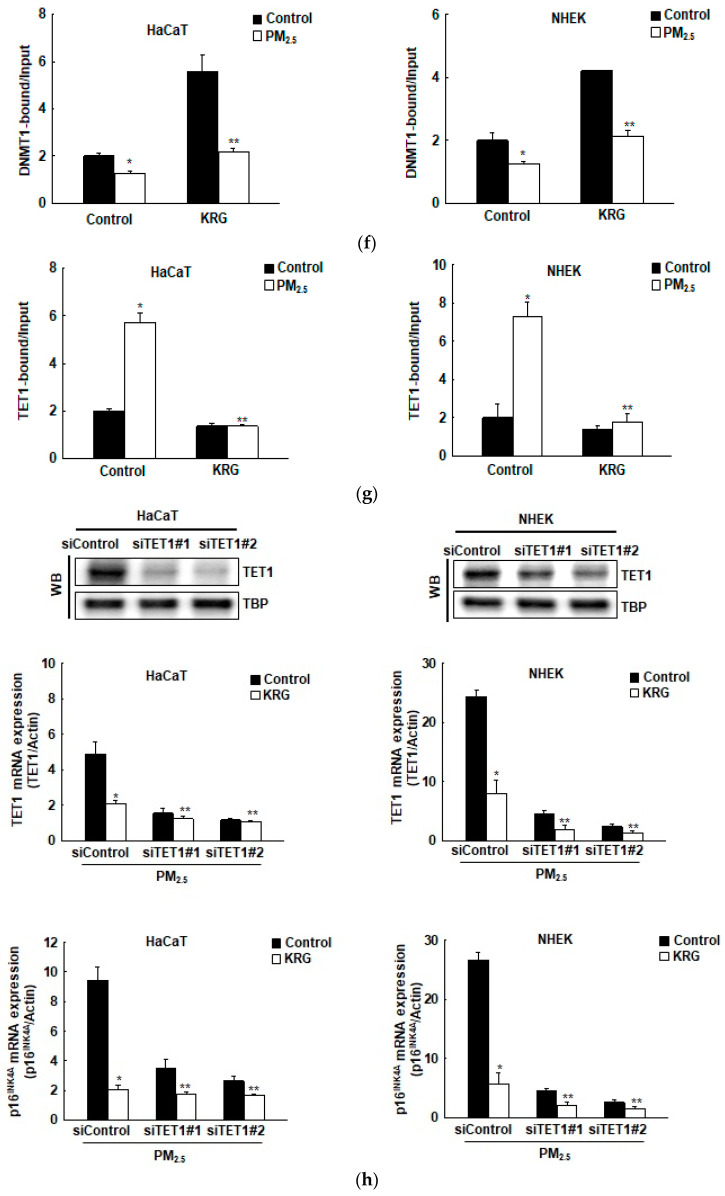
Attenuation of p16^INK4A^ expression in KRG-treated cells via an increase in DNA methylation. (**a**) The Alu element was used to normalize the quantitative methylation levels of the *p16^INK4A^* promoter region. (**b**) Analysis of the *p16^INK4A^* promoter region was assessed using bisulfite sequencing. Methylated cytosine is shown in black, whereas unmethylated cytosine is shown with white circles. The *p16^INK4A^* locus-targeted (chromatin immunoprecipitation (ChIP)-qPCR was carried out with the use of specified primer sets. (**c**) Electrophoresis of nuclear fractions was used to detect DNA methyltransferases (DNMTs) and ten-eleven translocations (TETs) via western blotting using specific antibodies. The loading control is represented here by the TATA-binding protein (TBP). (**d**,**e**) The nuclear location of (**d**) DNMT1 and (**e**) TET1 was determined by confocal microscopy after Alexa488-labeling (green) with the corresponding antibodies and staining with DAPI (blue). (**f**,**g**) ChIP assays using antibodies against (**f**) DNMT1 and (**g**) TET1 were performed and analyzed by qPCR. TET1 small interfering RNA (siRNA) was transfected into cells and then incubated for 24 h. (**h**) TET1 and p16^INK4A^ were detected by qRT-PCR. * *p* < 0.05 and ** *p* < 0.05 indicate significant differences with control cells and PM_2.5_-treated cells, respectively.

Our previous study demonstrated that *p16^INK4A^* is epigenetically controlled via the methylation of DNA during cellular senescence induced by PM_2.5_ [9]. To determine whether KRG is involved in the regulation of PM_2.5_-induced p16^INK4A^ transcription via epigenetic DNA methylation, we measured the *p16^INK4A^* promoter methylation status using qMSP and bisulfite sequencing analysis. The DNA methylation level of the *p16^INK4A^* promoter region (from –150 to +200 bp, including 35 CpG sites in the promoter of *p16^INK4A^*) significantly declined in the PM_2.5_-treated cells compared with that in the control cells; however, KRG increased the DNA methylation level in both of the cell types (Figure 3a). Subsequently, the DNA methylation status of the *p16^INK4A^* locus was examined with bisulfite sequencing, revealing that the PM_2.5_-treated cells had lower methylation levels than those of the control cells. However, KRG increased methylation in both of the cell types (Figure 3b). Therefore, KRG reduced the transcription of *p16^INK4A^* in the PM_2.5_-treated cells by attenuating epigenetic promoter methylation. In addition, western blot analyses showed that DNMT1, DNMT3A, and DNMT3B expression decreased in the PM_2.5_-treated cells but was increased by KRG (Figure 3c). Conversely, TET1, TET2, and TET3 expression increased in the PM_2.5_-treated cells but was reduced after applying the KRG treatment (Figure 3c). The expression of DNMTs and TETs by western blot analysis was confirmed using immunofluorescence (Figure 3d,e). We then assessed whether DNMT and TET could directly bind to the *p16^INK4A^* promoter region in both cell types by employing ChIP-qPCR analysis. The binding of DNMT1 to the *p16^INK4A^* promoter region decreased in the PM_2.5_-treated cells; however, KRG increased its binding in both cell types, which showed a similar pattern to that of protein expression (Figure 3f). The binding of TET1 to the *p16^INK4A^* locus increased in the PM_2.5_-treated cells but was decreased in both cell types by KRG (Figure 3g). Furthermore, TET1 siRNA decreased the expression of p16^INK4A^ mRNA in the PM_2.5_-treated cells, while KRG decreased PM_2.5_-induced p16^INK4A^ expression (Figure 3h). These results indicate that PM_2.5_-induced p16^INK4A^ expression involves the release of DNMT and the recruitment of TET participating in senescence induction. However, KRG attenuates these effects, leading to the inhibition of PM_2.5_-induced senescence-related gene transcription.

### 3.4. p16^INK4A^ Expression in KRG-Treated Cells Was Attenuated via Changes in Histone Methylation in Cellular Senescence Induced by PM_2.5_

**Figure 4:**
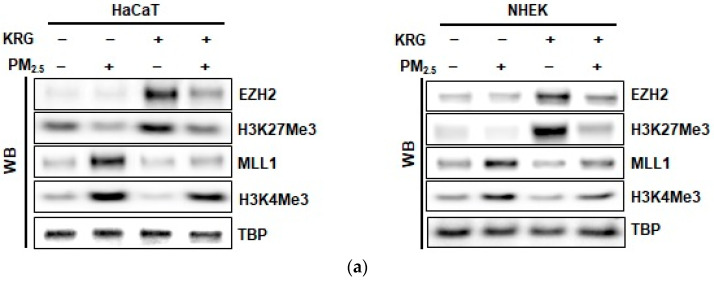

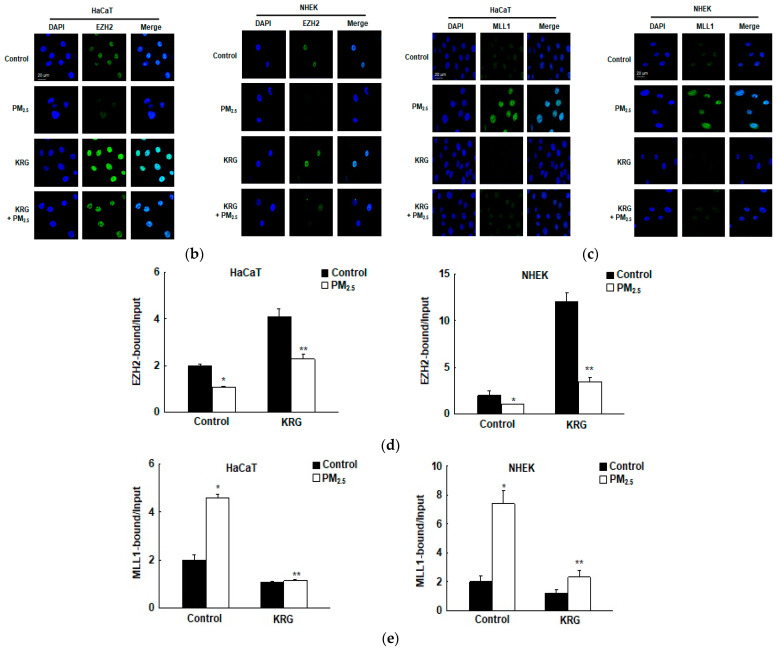

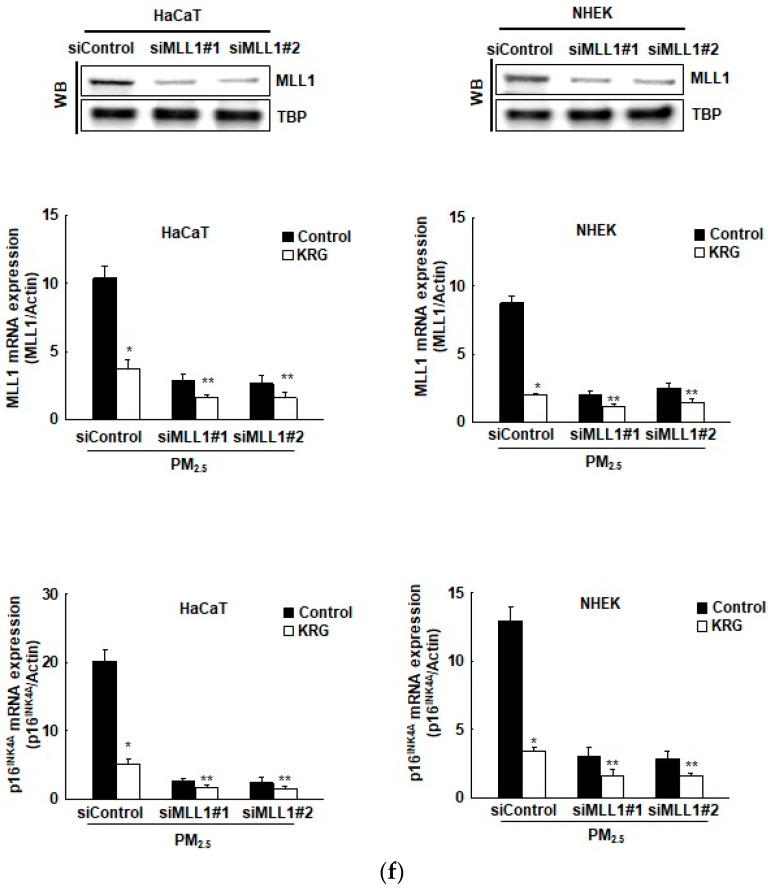
Attenuation of p16^INK4A^ expression in KRG-treated cells via changes in histone methylation. (**a**) Nuclear fractions were electrophoresed, and enhancers of zeste homolog 2 (EZH2), H3K27Me3, mixed-lineage leukemia 1 (MLL1), and H3K4Me3 were detected by western blotting with the corresponding antibodies. As indicated, TBP serves as the loading control. (**b**,**c**) The nuclear locations of (**b**) EZH2 and (**c**) MLL1 were determined by confocal microscopy after Alexa488-labeling (green) with the corresponding antibodies and staining with DAPI (blue). (**d**,**e**) ChIP assays using antibodies against (**d**) EZH2 and (**e**) MLL1 were performed and analyzed by qPCR. MLL1 siRNA was transfected into cells and incubated for 24 h. (**f**) Nuclear fractions were electrophoresed, and MLL1 was detected by western blotting with the corresponding antibodies. As indicated, TBP serves as the loading control. mRNA levels of MLL1 and p16^INK4A^ were detected by qRT-PCR. * *p* < 0.05 and ** *p* < 0.05 indicate significant differences with control cells and PM_2.5_-treated cells, respectively.

Our previous study demonstrated that histone methylation plays an important role in p16^INK4A^ epigenetic regulation [9]. To determine whether KRG is involved in epigenetic histone methylation of p16^INK4A^ expression induced by PM_2.5_, we detected the expression of HMT proteins using western blot analysis. EZH2, a transcriptional suppressor and a polycomb complex component with H3K27 methyltransferase activity, and its target protein, H3K27Me3, decreased after PM_2.5_ treatment; however, they were increased by KRG (Figure 4a). The expression of MLL1, a transcriptional activator with H3K4 methyltransferase activity, and its target protein (H3K4Me3) increased after PM_2.5_ treatment; however, they were decreased by KRG (Figure 4a). The expressions of EZH2 and MLL1 were confirmed by immunofluorescence analysis (Figure 4b,c). We assessed whether histone methyltransferase-related proteins could directly bind to the p16^INK4A^ locus in HaCaT and NHEK cells using ChIP-qPCR analysis. The binding of EZH2 to the p16^INK4A^ locus was decreased in PM_2.5_-treated cells; however, KRG increased their binding, which showed a similar pattern to that of protein expression (Figure 4d). The binding of MLL1 to the p16^INK4A^ locus increased in the PM_2.5_-treated cells but was decreased by KRG in both of the cell types (Figure 4e). In addition, MLL1 siRNA decreased the expression of p16^INK4A^ mRNA in the PM_2.5_-treated cells, and KRG decreased the PM_2.5_-induced p16^INK4A^ expression (Figure 4f). Collectively, these results indicate that the PM_2.5_-induced expression of p16^INK4A^ involves the recruitment of MLL1, whereas the release of EZH2 is linked to senescence induction. However, KRG attenuates these effects, leading to the inhibition of p16^INK4A^ transcription induced by PM_2.5_.

### 3.5. p16^INK4A^ Expression in KRG-Treated Cells Was Attenuated via Changes in Histone Acetylation and Deacetylation in Cellular Senescence Induced by PM_2.5_

**Figure 5:**
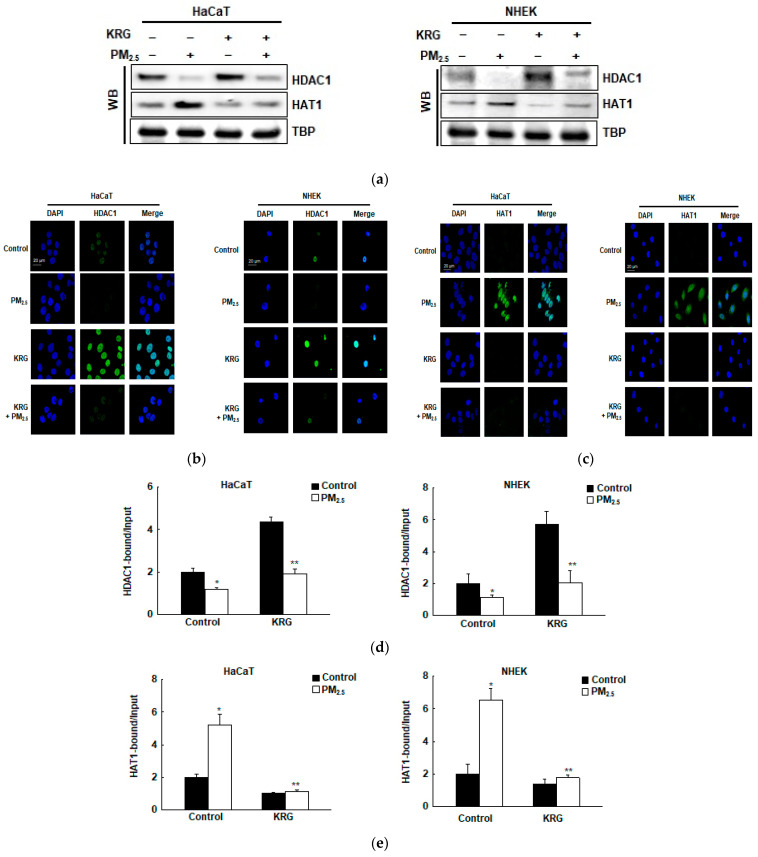

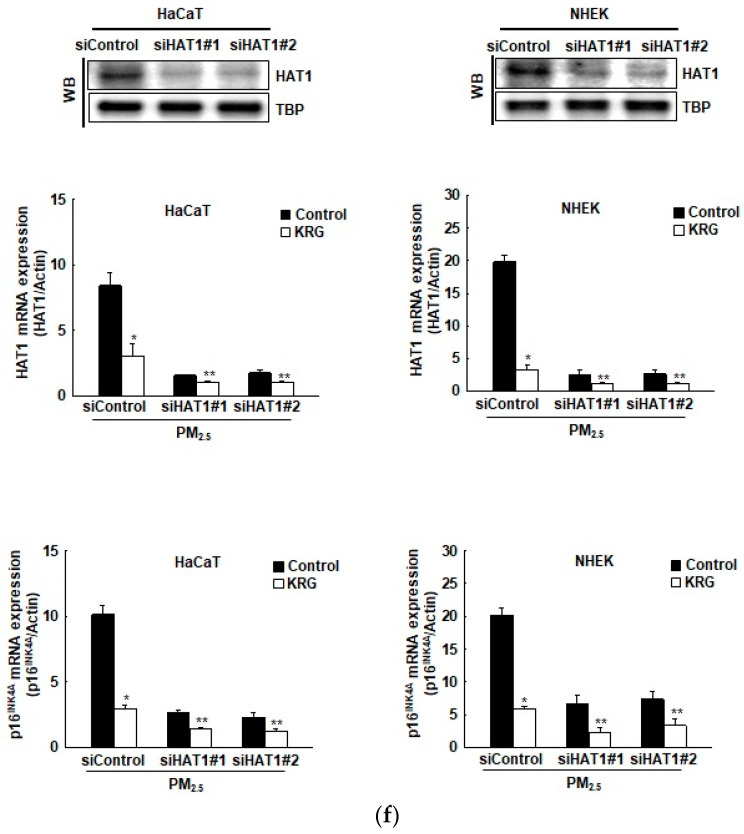
Attenuation of p16^INK4A^ expression in KRG-treated cells via changes in histone acetylation and deacetylation. (**a**) Nuclear fractions were electrophoresed, and histone deacetyltransferase 1 (HDAC1) and histone acetyltransferase 1 (HAT1) were detected using western blotting with the corresponding antibodies. As indicated, TBP serves as the loading control. (**b**,**c**) The nuclear locations of (**b**) HDAC1 and (**c**) HAT1 were determined by confocal microscopy after Alexa488-labeling (green) with the corresponding antibodies and staining with DAPI (blue). (**d**,**e**) ChIP assays using antibodies against (**d**) HDAC1 and (**e**) HAT1 were performed and analyzed by qPCR. HAT1 siRNA was transfected into cells and incubated for 24 h. (**f**) Nuclear fractions were electrophoresed, and HAT1 was detected using western blotting with the corresponding antibodies. As indicated, TBP serves as the loading control. The mRNA levels of HAT1 and p16^INK4A^ were detected by qRT-PCR. * *p* < 0.05 and ** *p* < 0.05 indicate significant differences with control cells and PM_2.5_-treated cells, respectively.

Many studies have shown that histone acetylation plays an important role in *p16^INK4A^* epigenetic regulation [25]. To determine whether KRG is involved in the epigenetic histone acetylation of p16^INK4A^ expression induced by PM_2.5_, we detected the expression of histone acetylation and deacetylation proteins using western blot analyses. The expression of the transcriptional repressor HDAC1 decreased in the PM_2.5_-treated cells with deacetylase activity; however, it was increased by KRG (Figure 5a). The transcriptional activator HAT1 increased in the PM_2.5_-treated cells with acetyltransferase activity; however, treatment with KRG decreased it (Figure 5a). The expressions of HDAC1 and HAT1 were confirmed using immunofluorescence (Figure 5b,c). Furthermore, we assessed whether histone acetyl-related proteins could directly bind to the *p16^INK4A^* locus using ChIP-qPCR analysis. The binding of HDAC1 to the *p16^INK4A^* locus was decreased in the PM_2.5_-treated cells but was increased by KRG in both cell types, with a pattern comparable to that of protein expression (Figure 5d). The binding of HAT1 to the *p16^INK4A^* locus increased in the PM_2.5_-treated cells but was decreased by KRG in both cell types (Figure 5e). In addition, HAT1 siRNA decreased the expression of p16^INK4A^ mRNA in the PM_2.5_-treated cells, while KRG decreased the PM_2.5_-induced p16^INK4A^ expression (Figure 5f). Collectively, these results indicate that p16^INK4A^ expression induced by PM_2.5_ involves the recruitment of HAT1, while the release of HDAC1 is associated with cellular senescence induction. However, KRG has been shown to weaken this effect and induce the inhibition of p16^INK4A^ transcription through increased histone deacetylation.

## 4. Discussion

PM_2.5_ has been reported to cause skin diseases such as allergies, inflammatory dermatitis, and skin senescence, resulting in harmful effects on the skin. We have previously reported that PM_2.5_ induced an increase in oxidative stress through the aryl hydrocarbon receptor-ROS pathway and promoted the binding of TET1 and MLL1, instead of DNMT1 and EZH2, to the *p16^INK4A^* promoter region. This binding induced the expression of p16^INK4A^, ultimately inducing human epidermal keratinocytes senescence [9].

KRG has been reported to have more pharmacological efficacy than that of fresh and white ginseng [37]. About 40 types of ginsenosides, including Rb1, Rb2, Rc, Rd, Re, and Rg1 as main bioactive ingredients, have been identified in KRG [37,38]. All compounds exhibit their own biological activity, such as anticancer, anti-inflammatory, antioxidant, antibacterial, antiviral, and antifungal effects, but when mixed or combined, they can produce additive, synergistic, or increased effects [38,39,40,41,42].

Many studies have been conducted on the pharmacological properties of KRG with regard to skin senescence [43,44,45]. KRG has been shown to prevent endothelial senescence by downregulating the NF-κB/miRNA-155-5p/eNOS pathway [43]. In addition, the enzyme-modified ginseng extract from KRG has shown protective effects against ultraviolet-B-induced skin senescence in human skin fibroblasts [46]. Furthermore, several studies have explored the anti-senescence effects of ginsenosides, the main component of KRG [47,48,49,50]. For instance, ginsenoside Rb1 alleviates oxidative low-density lipoprotein-induced vascular endothelium senescence via the SIRT1/beclin-1/autophagy axis [47]. Additionally, ginsenoside Rg3 inhibits the senescence of prostate stromal cells through the down-regulation of interleukin 8 expression [48].

However, total extracts of ginseng are more beneficial and effective than single ginsenosides or combinations of specific ginsenosides [42]. Here, we determined the anti-senescent efficacy of KRG and the underlying molecular mechanism involving the epigenetic regulation of p16^INK4A^, a senescence sensor, in PM_2.5_-induced senescence. Our results have shown that KRG decreases the cellular senescence phenotypes associated with PM_2.5_-induced oxidative stress, including flattened and enlarged cell shapes, SAHF-like chromatin foci, and β-galactosidase activity in skin keratinocytes.

As a CDK4/CDK6 inhibitor, p16^INK4A^ plays a vital role in senescence formation. The p16^INK4A^ protein is relatively stable, and its expression is mainly controlled at the transcriptional level. Abnormal p16^INK4A^ hypermethylation is found in most tumors and reduces gene expression [51,52,53]. In mammalian cells, DNMTs maintain global and gene-specific de novo DNA methylation [24]. TETs have the ability to reverse this methylation process. When cells were exposed to PM_2.5_ in this study, TET1 replaced DNMT1 in the *p16^INK4A^* promoter region, promoting *p16^INK4A^* transcription. KRG pre-treatment in the cells that were treated with PM_2.5_ reversed the altered expressions of DNMTs and TETs. In addition, KRG decreased TET1 binding to the *p16^INK4A^* locus and increased DNMT1 binding, thereby reversing the increase in p16^INK4A^ expression and preventing cellular senescence.

Histone methylation via EZH2 and MLL1 is also vital for gene transcription. A methyltransferase EZH2 binds to the *p16^INK4A^* locus, inhibiting *p16^INK4A^* transcription. Conversely, the *p16^INK4A^* locus binding of MLL1 induces transcription during replication and premature senescence [54,55]. In the presence of PM_2.5_, MLL1 replaced EZH2 in the skin keratinocytes, thereby promoting the transcription of *p16^INK4A^*. However, KRG suppressed this effect. In addition to EZH2 and MLL1, the modification of histone acetylation via HAT or HDAC plays an important role in gene transcription [25,56]. In our study, PM_2.5_ induced p16^INK4A^ expression via histone acetylation with HAT1. However, KRG reversed p16^INK4A^ expression via histone deacetylation with HDAC1.

Considering these results, we conclude that p16^INK4A^ expression induced by PM_2.5_ is suppressed by KRG via the epigenetic regulation of DNA and histones, which results in the inhibition of cellular senescence. As KRG inhibited the p16^INK4A^ expression induced by PM_2.5_ through epigenetic regulation, it can be inferred that KRG protects the skin against PM_2.5_ exposure and reduces skin senescence. However, it remains unclear how other epigenetic regulation processes, such as those involving microRNA, are regulated during cellular senescence induced by PM_2.5_, and how KRG executes epigenetic regulation process of microRNA modulated by PM_2.5_. Hence, further studies are needed in order to determine other epigenetic regulation processes affecting cellular senescence.

## Data Availability

The data presented in this study are available within this manuscript.
